# A kaleidoscopic view of extracellular vesicles in lysosomal storage disorders

**DOI:** 10.20517/evcna.2022.41

**Published:** 2022-12-30

**Authors:** Charlotte V. Hegeman, Olivier G. de Jong, Magdalena J. Lorenowicz

**Affiliations:** ^1^Department of Pharmaceutics, Utrecht Institute for Pharmaceutical Sciences (UIPS), Utrecht University, Universiteitsweg 99, Utrecht 3584 CG, The Netherlands.; ^2^Regenerative Medicine Center, Uppsalalaan 8, Utrecht 3584 CT, The Netherlands.; ^3^Biomedical Primate Research Centre, Lange Kleinweg 161, Rijswijk 2288 GJ, The Netherlands.; **^#^** Authors contributed equally.

**Keywords:** Lysosomal storage disorders, extracellular vesicles, autophagy, therapeutics, diagnostics

## Abstract

Extracellular vesicles (EVs) are a heterogeneous population of stable lipid membrane particles that play a critical role in the regulation of numerous physiological and pathological processes. EV cargo, which includes lipids, proteins, and RNAs including miRNAs, is affected by the metabolic status of the parental cell. Concordantly, abnormalities in the autophagic-endolysosomal pathway, as seen in lysosomal storage disorders (LSDs), can affect EV release as well as EV cargo. LSDs are a group of over 70 inheritable diseases, characterized by lysosomal dysfunction and gradual accumulation of undigested molecules. LSDs are caused by single gene mutations that lead to a deficiency of a lysosomal protein or lipid. Lysosomal dysfunction sets off a cascade of alterations in the endolysosomal pathway that can affect autophagy and alter calcium homeostasis, leading to energy imbalance, oxidative stress, and apoptosis. The pathophysiology of these diseases is very heterogenous, complex, and currently incompletely understood. LSDs lead to progressive multisystemic symptoms that often include neurological deficits. In this review, a kaleidoscopic overview will be given on the roles of EVs in LSDs, from their contribution to pathology and diagnostics to their role as drug delivery vehicles. Furthermore, EV cargo and surface engineering strategies will be discussed to show the potential of EVs in future LSD treatment, both in the context of enzyme replacement therapy, as well as future gene editing strategies like CRISPR/Cas. The use of engineered EVs as drug delivery vehicles may mask therapeutic cargo from the immune system and protect it from degradation, improving circulation time and targeted delivery.

## INTRODUCTION

**Figure 1:**
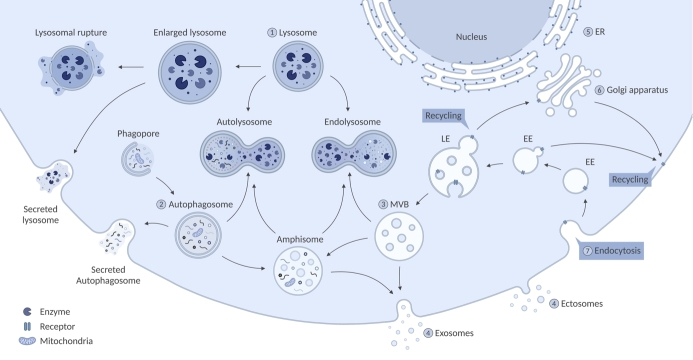
A schematic illustration of different pathways affected in LSDs. (1) In LSDs, a single gene mutation causes the deficiency of a lysosomal protein or lipid. The defect leads to gradual accumulation of macromolecules, inhibition of other lysosomal components, and enlargement of the lysosome which can be followed by lysosomal rupture and secretion. Furthermore, dysfunction of the lysosome and its altered calcium (Ca^2+^) homeostasis leads to impaired fusion with cellular structures including autophagosomes and endosomes/multivesicular bodies (MVBs). (2) The accumulation of autophagosomes can potentially lead to secretion of the organelle to clear its obstruction. Additionally, autophagy is downregulated by hyperactivation of mTOR in some LSDs (MPS-I, MPS-VI, MPS-VII, and Gaucher Disease). Subsequently, impaired or inhibited autophagy results in mitochondrial dysfunction that can lead to energy imbalance, apoptosis, and inflammation. (3) There is a balance between degradation and EV release, which is highly dependent on the faith of MVBs. (4) Impaired fusion of MVBs with the lysosome can lead to fusion of MVBs with the plasma membrane, which causes increased secretion of EVs (Niemann-Pick type C, Sialidosis, Krabbe and Fabry Disease). Hence, inhibition of autophagy and subsequential increase of EV secretion might be an alternative route for LSD cells to clear obstruction of the accumulating molecules to naturally improve the cellular phenotype. (5) In multiple sphingolipid storage disorders (Gaucher Disease, GM1 and GM2 Gangliosidoses, and Niemann-Pick type A) Endoplasmic reticulum (ER) calcium homeostasis is perturbed, which leads to elevated Ca^2+^ in the cytosol. (6) Dysfunction of the Golgi apparatus has been reported for most lipid storage disorders and MPS IIIB. This includes altered morphology and accumulation of large storage bodies.

Lysosomal storage disorders (LSDs) include over 70 rare heritable (inborn) errors of metabolism. In LSDs, a single gene mutation causes deficiency of a lysosomal protein or lipid, which leads to gradual accumulation of undigested macromolecules and negatively affects lysosomal function. Because lysosomal hydrolases are involved in the stepwise degradation, primary substrate accumulation in lysosomes gradually leads to secondary accumulation of upstream substrates within the same catabolic pathway. The accumulation leads to enlargement of the organelle and functional inhibition of genetically correct enzymes and proteases which further limits their processing abilities^[1-3]^. This sets off a cascade of alterations in the endolysosomal pathway due to impaired fusion that can affect autophagy, but also in other organelles including the endoplasmic reticulum and mitochondria^[4]^. This may further lead to altered calcium homeostasis, oxidative stress, energy imbalance, and apoptosis^[5,6]^. The degree of the effect on these cellular processes highly depends on the type of LSD and its specific genetic mutation^[7]^. The complex interplay of the cellular pathways is shown in Figure 1. The cellular impairments seen in LSDs can lead to cell death and cause progressive multisystemic symptoms that often include visceromegaly, bone abnormalities, cardio-respiratory problems, and neurodegeneration^[8,9]^.

Around 70% of the LSDs are enzyme deficiencies and can be further subclassified based on their storage material, such as Glycoproteinoses, Mucopolysaccharidoses, and Lipidoses. Other subgroups of LSDs include deficiencies in integral membrane proteins, transporters and proteins involved in trafficking, regulation, or post-translational modification. Despite the classification, LSDs are clinically very heterogeneous, even within the same subtype. The symptoms depend on the specific genetic mutation and to which degree this affects the lysosomal protein or lipid, as well as the spatial and temporal accumulation of macromolecules^[7]^. While individual LSDs are rare, collectively, they have an estimated incidence of 1 in 5000-8000^[10-12]^. All LSDs that will be described in this review are listed in Table 1.

**Table 1:** Described Lysosomal Storage Disorders

**Disease (gene)** Eponyms and/or types	**Primary deficiency**	**Accumulated molecules**	**Approved therapies**
**Sphingolipidoses**			
**Fabry Disease** ^a^ ** (GLA)**	α- Galactosidase A	Globotriaosylceramide	ERT and CT
**Farber Lipogranulomatosis** ^a^ ** (ASAH1)**	Acid ceramidase	Ceramide	S/S
**Gaucher Disease** ^a^ ** (GBA)** Type I, II^b^, III^b^, perinatal lethal form^b^ and cardiovascular^[13]c^	β Glucocerebrosidase/β- glucosidase	Glucocerebroside/ glucosylsphingosine	ERT and SRT (types I, II and III), none (perinatal lethal form)
**GM2 Gangliosidosis ** Tay-Sachs Disease^c^ (HEXA)	β- Hexosaminidase	GM2 ganglioside, glycosphingolipids and oligosaccharides	S/S
**Globoid Cell Leukodystrophy** ^a^^,^^b ^ **(GALC)** Krabbe Disease	Galactosylceramidase	Galactocerebroside and psychosine	HSCT (infantile onset) and S/S
**Metachromatic Leukodystrophy** ^a^^,^^b ^ **(ARSA and PSAP)** MLD	Arylsulfatase A Sphingolipid activator protein SAP-B	Sulfatides	HSCT
**Niemann-Pick Disease** ^a^ ** (SMPD1)** Type A^b^ and B	Sphingomyelin Phosphodiesterase	Sphingomyelin	S/S
**Mucopolysaccharidosis (MPS)**			
**MPS III** ^a^^,^^b^^,^^d^ III-A Sanfilippo A (SGSH) III-C Sanfilippo C (HGSNAT)	N- Sulfoglucosamine sulfohydrolase Heparan- α-glucosaminide- N- acetyltransferase	HS (KS)	S/S
**MPS IV** ^a^ IV-A Morquio syndrome A (GALNS)	N-acetylgalactosamine- 6-sulfatase	KS, C6S	ERT (IV-A) and S/S (IV-B)
**MPS VII** ^a^^,^^b ^ **(GUSB)** Sly Disease	β- Glucuronidase	DS, HS, C4S, C6S (KS)	ERT
**Glycogen Storage Disorders (GSD)**			
**GSD II** ^a^ ** (GAA)** Pompe Disease	Lysosomal α- glucosidase/acid maltase	Glycogen	ERT
**Glycoproteinoses**			
**Sialidosis** ^a^ ** (NEU1)** Type I, cherry-red spot myoclonus Type II^b^, Mucolipidosis I	Neuraminidase-1	Sialylated oligosaccharides and glycopeptides Sialylated oligosaccharides and glycopeptides, LAMP1 and amyloid precursor protein	S/S
**Integral Membrane Protein Disorders**			
**Cystinosis (CTNS)**	Cystinosin	Cystine	SMT
**Niemann-Pick Disease** C1 (NPC1) C2^b^ (NPC2)	NPC intracellular cholesterol transporter 1 NPC intracellular cholesterol transporter 2	Cholesterol and sphingolipids	SRT
**Neuronal Ceroid Lipofuscinoses (NCL or CLN), Batten Disease**			
**CLN2** ^b^ ** (TPP1)** Jansky-Bielschowsky Disease	Tripeptidyl peptidase 1	Subunit c of mitochondrial ATP synthase	ERT

CT: Chaperone therapy; C4S: chondroitin 4-Sulfate; C6S: chondroitin 6-sulfate; DS: dermatan sulfate; ERT: enzyme replacement therapy; HS: heparan sulfate; HSCT: hematopoietic stem cell therapy; KS: keratan sulphate; LAMP1: lysosome-associated membrane glycoprotein 1; SMT: small-molecule therapy; SRT: substrate reduction therapy; S/S: symptomatic and supportive therapy. ^a^Denotes enzyme deficiency. ^b^Denotes diseases that involve the Central Nervous System (CNS). ^c^Type depends on specific mutation^[14]^. ^d^Denotes disorders with severe and attenuated forms that result in a phenotype spectrum^[7,15]^.

To date, the most established treatment option for LSDs is enzyme replacement therapy (ERT)^[16]^. ERT is available for several enzyme deficiencies, where it can improve clinical symptoms and slow down disease progression. Unfortunately, ERT comes with serious limitations that include the development of neutralizing antibodies against the enzyme, low plasma half-life, inability to cross the blood-brain barrier (BBB), and high costs. Furthermore, patients treated with ERT are left with residual disease due to its poor biodistribution, which fails to effectively target certain organs^[17-19]^. Besides ERT, hematopoietic stem cell transplantation (HSCT) is available for a very limited group of LSDs, including MPS-IH, MPS-II, α-Mannosidosis, MLD, and Krabbe Disease. To improve its effects and survival of patients, early diagnosis and early treatment with HSCT are critical. The effect of stem cell transplantation is based on the production of the missing enzyme by the healthy donor cells that will allow cross-correction^[20-25]^. However, HSCT is not fully curative and unable to completely halt disease in hard-to-reach tissues. This is often the case for connective tissue, resulting in progression of skeletal malformations, corneal clouding, valvular dysfunction, as well as inconsistent results for joint, tendon, and ligament stiffness^[15]^. Several other novel strategies have been developed over the years, including substrate reduction therapy (SRT), pharmacological chaperone therapy (PCT), and gene therapy^[26-28]^. For most LSDs, the only solution remains symptomatic treatment and supportive care^[7]^. A cure that will be more broadly applicable to treat more LSDs, including those with severely affected neurological manifestations, has yet to be discovered.

Post-treatment residual disease in connective tissues has drawn attention towards multipotent mesenchymal stem cells (MSCs), which have the ability to differentiate into multiple lineages of bone, cartilage, and fat. Moreover, MSCs are characterized by their intrinsic self-renewal capacity and their capability of immunomodulation. Their transplantation is widely tested in clinical trials and considered safe^[29,30]^. In comparison to hematopoietic stem cells (HSC), MSCs secrete significantly higher levels of mucopolysaccharidosis (MPS)-related enzymes^[31]^. Thus, MSCs might be able to improve skeletal malformations and neurological deficits in LSD patients at an early stage of disease development^[29,32]^. Several studies with MSCs derived from various tissue sources have been performed in mice models for different LSDs. In these studies, improvement in motor function, decreased inflammation and apoptosis were reported post-injection of the cells into different parts of the brain^[5]^. To date, intravenous injection of MSCs has only been tested on patients with Metachromatic Leukodystrophy (MLD); this showed promising results regarding its safety and improvement of stabilization on neurological manifestations^[33,34]^. Currently, a clinical trial is assessing the safety of human placental-derived MSCs (phase I) in multiple other LSDs (ID: NCT01586455). However, increasing evidence suggests that the therapeutic efficiency of MSC therapy is not dependent on the engraftment of MSCs or their capability to differentiate after transplantation. Most likely, the mode of action of MSCs relies on their paracrine signaling including extracellular vesicles (EVs) to support surrounding tissues^[35]^.

Over the last two decades, attention has been drawn towards EVs as important mediators of intercellular communication. EVs are a heterogeneous population of stable lipid membrane particles and play a critical role in the regulation of numerous physiological and pathological processes^[36]^. EVs capture the metabolic status of the cell^[37]^, which qualifies them as potential sources of biomarkers for LSDs. Furthermore, EVs are biocompatible, low immunogenic and can traverse multiple biological barriers. For instance, several studies have indicated that EVs are able to cross the BBB^[38,39]^. Additionally, bioengineering approaches can modify EVs to load specific cargo or target specific sites^[40]^. This makes EVs of high interest as both therapeutic agents and drug delivery vehicles, which could open doors to the development of novel treatments for LSDs including those with neurological deficits. In this review, we will provide an overview of the current knowledge and potential of EVs in unraveling LSDs, their diagnostics, treatment, and potential future drug delivery for gene correction strategies. For an overview of the existing synthetic nanotechnology-based approaches that have been explored as delivery vehicles in the treatment of LSDs, we refer the reader to the reviews of Abasolo *et al.*, Schuh* et al.*, and Del Grosso* et al.*^[41,42,43]^*.*

## THE BIOGENESIS OF EXTRACELLULAR VESICLES

EVs are a heterogeneous population of small lipid membrane vesicles that are secreted by all cells, and have been observed in all body fluids^[36]^. EVs are conventionally classified into subtypes based on their biogenesis: exosomes and ectosomes. Exosomes (40-140 nm) are a part of the endo-lysosomal system and form through intraluminal vesicle (ILV) formation by inward membrane budding in the late endosome. This process involves the endosomal sorting complexes required for transport (ESCRT) machinery, tetraspanins and lipid-dependent interactions, which ultimately leads to the formation of multivesicular bodies (MVBs)^[44]^. MVBs can release their intraluminal vesicles upon fusion with the plasma membrane into the extracellular space, after which they are referred to as exosomes. Ectosomes, also referred to as microvesicles (50-1000 nm), directly bud off the plasma membrane^[45]^. Clear classification of EVs remains a challenge due to their heterogenous size, cargo, and lack of specific biomarkers to distinguish the EV subtypes^[46,47]^.

EVs are known for their contribution to cell-to-cell communication by transferring cargo such as RNA, including mRNA and long noncoding RNAs, lipids, and proteins including lysosomal enzymes^[48-50]^. Over the last decade, it has become clear that EV cargo reflects the status of the secreting cell and its tissue origin^[37,51]^. Cargo transportation through EVs plays a critical role in the regulation and progression of numerous physiological and pathological processes^[36]^. The contribution of EVs to pathology has been shown for several neurological disorders that have been associated with LSDs or other pathologies related to endolysosomal dysfunction, such as Parkinson’s Disease (PD)^[52]^, Alzheimer’s Disease^[53]^, and Huntington’s Disease^[54,55]^. Since these neurological disorders are currently not classified as classical LSDs, describing the endolysosomal involvement in the pathology of these disorders is beyond the scope of this review. For more in-depth information on these diseases, we refer the readers to the reviews of Navarro-Romero *et al.* and Almeida *et al.*^[56,57]^*. *In these neurological disorders, EVs are thought to contribute to inflammation and seem to sequester and spread the accumulating pathogenic proteins^[53,58]^. This suggests that EVs may contribute to the altered physiology in LSDs as well^[59]^. This makes EVs a potentially useful source of information to further determine the altered processes in LSDs as biomarkers for diagnostics and disease progression, including their location^[51,60]^.

As EVs hold the ability to traverse multiple biological barriers and can be modified to avoid uptake by the mononuclear phagocytic system, EVs are potential therapeutic agents and drug delivery vehicles for LSDs, due to their biocompatibility and low immunogenicity^[61-63]^. Adaptation of EVs through bioengineering approaches could further optimize their delivery potential and therapeutic value by targeted loading of therapeutic components through fusion proteins, or to target EVs to specific tissues^[40,64]^. This strategy could actively load ERT into EVs, which could reduce neutralizing antibodies against the enzyme, improve its plasma half-life, and facilitate delivery towards the nervous system by crossing the BBB^[38]^. As such, EVs have the potential to both improve existing treatment and lead to new therapeutic options for LSD patients in the future, including those with severe neurological deficits.

## EV RELEASE IN LSDs: A NEW ROUTE IN UNDERSTANDING DISEASE PATHOLOGY

Lysosomes are essential organelles in the degradation of macromolecules delivered by endocytosis or autophagocytosis. The lysosomal characteristic acidic lumen includes over 60 hydrolases that can return molecules to catabolites, thereby allowing recycling of these molecules. These functions allow lysosomes to play a fundamental role in multiple cellular processes including autophagy and its regulation, cellular homeostasis, cell signaling, mitochondrial function and vesicle fusion^[65,66]^. Several of these processes are also implicated in EV release and uptake, which are both parts of the endolysosomal pathway. As described before, the fusion of MVBs with the plasma membrane leads to EV release. Alternatively, MVBs can fuse with both lysosomes and autophagosomes, leading to degradation of MVBs. Moreover, both studies on ESCRT proteins and autophagy modulators show a tight relationship between autophagy and MVB biogenesis, thereby determining the faith of its ILVs and, subsequently, of EVs^[44]^. The lysosomal dysfunction in LSDs does not make it difficult to envision its effects on both EV release and uptake, which will be discussed in this section.

Autophagy is a multi-step process where damaged organelles and molecular aggregates are degraded and recycled. The process starts with the formation of autophagosomes that can fuse with early and late endosomal vesicles, such as MVBs, to form amphisomes, to eventually fuse with lysosomes^[4,7]^. Under homeostatic conditions, cells maintain a constitutive basal level of autophagy; however, the process can be induced in response to cellular and metabolic stress^[67]^. In several LSDs, it has been shown that lysosomal dysfunction leads to the accumulation of autophagosomes and autophagic substrates, such as damaged mitochondria^[4,68,69]^. However, accumulation of autophagosomes does not necessarily indicate an increased induction of autophagy as this increase could also be caused by blockage of fusion with the lysosome. Nevertheless, the autophagy pathway in LSDs is disrupted, which could either mean activation or inhibition of autophagy, depending on the LSD type. For instance, in MPS-I, MPS-VI, MPS-VII, and Gaucher Disease, hyperactivation of mTOR, a negative regulator of autophagy, has been shown^[69,70]^. In this study, mTOR hyperactivation increased phosphorylation of the UVRUG protein, which led to reduced autophagosome maturation and reduced fusion with the lysosome^[69]^. Moreover, activated mTOR can phosphorylate transcription factor EB (TFEB), which inhibits its translocation to the nucleus and, with that, lysosomal biogenesis and function^[8]^. Thus, there might be a vicious cycle in play where lysosomal accumulation inhibits the induction of autophagy, perhaps to prevent further accumulation-induced damage. The complex interplay of autophagy and the endolysosomal pathways is shown in Figure 1.

There is a balance between autophagy and EV release, which is highly dependent on the faith of MVBs. It has been shown that in hepatic stellate cells, activation of mTOR signaling induced the release of exosomes by inhibiting autophagy. Furthermore, the activation of mTOR can stimulate release of microvesicles through activation of ROCK1 signaling^[71]^. The hyperactivation of mTOR in some LSDs could play a vital part in the secretion of EVs by these cells. In a study by Bartolomeo *et al.*, it was shown that mTOR hyperactivity significantly reduced the formation of Phosphatidylinositol-3-phosphate (PI3P)^[69]^. This may influence EV release considering that PI3P is required in vesicle and (auto)phagosome fusion with lysosomes. As PI3P reduction may limit the fusion between MVBs and autophagosomes, this could potentially shift the faith of MVBs towards fusion with the plasma membrane and secretion of EVs instead, to limit accumulation and maintain homeostasis. Furthermore, PI3P is involved in ESCRT protein recruitment and cargo organization, and serves as a substrate for PI(3,5)P_2_ formation^[72,73]^. Therefore, the diminished presence of PI3P through mTOR hyperactivation could also lead to reduced PIP_2_ formation. The absence of PIP_2 _has been shown to increase EV secretion^[74]^. In addition, reduction of PI3P formation promoted secretion of atypical exosome containing undigested lysosomal components in neuronal disorders with lysosomal dysfunction closely related to Niemann-Pick type C^[73,75]^. A study by Strauss *et al.* confirmed increased EV release in Niemann-Pick type C cells^[76]^. Table 2 gives an overview of all studies describing the EV release by LSD cells. In Niemann-Pick type C, loss of function of a late endosomal membrane protein leads to accumulation of free cholesterol and sphingolipids. Strauss *et al.* showed a flotillin-dependent pathway of EV secretion as an alternative to secrete free cholesterol to ameliorate its storage within cells^[76,77]^. Moreover, increased EV release has been shown in several enzyme deficiencies, including Sialidosis, Krabbe and Fabry Disease^[68,78,79]^. Hence, inhibition of autophagy and subsequential increase of EV secretion might be an alternative route for LSD cells to clear obstruction of the accumulating molecules to improve the cellular phenotype.

**Table 2:** The secretion of extracellular vesicles in lysosomal storage disorder models

**LSD**	**Disease model**	**EV isolation method**	**Major results**	**Reference**
**Fabry Disease**	*In vitro* CRISPR/Cas9 altered human embryonic stem cells differentiated into cardiomyocytes	Total Exosome Isolation Reagent (Thermo Fisher Scientific)	> Downregulation of proteins related to EV transportation, secretion, and exocytosis (Rab GTPases: Rab-11, GDIR2, VPS36 and VTI1A) > Impairment of the autophagic flux and protein turnover, which increased reactive oxygen species, apoptosis, and necrosis > Increased production and secretion of exosomes	[68]
**Farber Lipogranulo-matosis (Partial model)**	Asah1^fl/fl^/Smooth Muscle (SM)^cre^ mice	Centrifugation (4 °C): 300 *g*, 10 min Filter 0.22 µm filters to 100,000 *g*, 90 min *Washing step* 100,000 *g*, 90 min	> Acid ceramidase (Ac) contributes to the development of arterial medial calcification (AMC) in the aorta and increases osteogenic markers > Decreased colocalization of MVBs with lysosomes and upregulation of sEV markers was seen in coronary arterial smooth muscle cells (CASMCs) of vitamin D treated Asah1^fl/fl^/SM^Cre^ mice; The same effect was observed in phosphate (Pi) treated CASMCs *in vitro* > Untreated Asah1^fl/fl^/SM^Cre^ CASMCs show increased sEV release, whereas Pi treated Asah1^fl/fl^/SM^Cre^ CASMCs show even more sEV release and increased MVB formation > Ac gene deletion remarkably decreased ML-SA1 induced Ca^2+^ release through TRPML1 channels	[80]
**Krabbe Disease**	*In vitro* Primary erythrocytes and oligodendrocytes from Twitcher mice-pups	Centrifugation: 100 *g*, 10 min at RT 21.000 *g*, 20 min at 4 °C	> Psychosine accumulation introduces focal regions of rigidity in the plasma membrane, which affects curvature and facilitates increased budding and shedding of 0.5-4 μm EVs in erythrocytes and oligodendrocytes	[79]
	*In vivo* Twitch mice (GALC^-/-^)	Centrifugation: 300 *g*, 10 min (P1) 2.000 *g*, 10 min (P2) 10,000 *g*, 30 min (P3) 100,000 *g*, 90 min (P4) *Sucrose gradient* 200,000 *g*, 16 h *Washing step fractions* 100,000 *g*, 90 min	> At early disease stage, an increased number of brain-derived EVs was found, and this was decreased at a late stage > Significant increase in psychosine, myelin proteolipids and myelin basic proteins in brain-derived EVs, which further increased parallel to disease development > Treatment with ceramide production inhibitor GW4869 significantly decreased the number of brain-derived EVs, which accelerated the acquisition of the final disease state	[81]
**MLD**	*In vivo* ASA^-/-^ mice (cortex) *In vitro* Primary Neural Precursor (NP) and glial cells	Centrifugation (4 °C): 300 *g*, 10 min 2000 *g*, 10 min 10,000 *g*, 30 min 100,000 *g*, 70 min *Wash step* 100,000 *g*, 70 min *Sucrose gradient* 200,000 *g*, 16 h *Washing step fractions* 100,000 *g*, 70 min Filtration (0.22 μm) Centrifugation: 100,000 *g*, 90 min *Washing step* 100,000 *g*, 90 min	> ASA^-/-^ Cells have a defective PDGFRα pathway: Reduced PDGFRα protein levels and failure to phosphorylate AKT > ASA^-/-^ glia, NP and cortex-derived exosomes contain significantly increased PDGFRα levels > Enzyme correction of ASA^-/-^ NPs normalized PDGFRα levels, led to re-localization of the receptor in detergent-resistant membrane domains, increased AKT phosphorylation, normalized the production of oligodendrocytes and reduced exosomal shedding > PDGFRα is secreted* in vivo* through exosomes during the peak of myelination	[82]
**Sialidosis** **(type I and II)**	*In vitro* Neu1^-/-^ mice myofibroblasts Human skin fibroblasts	Centrifugation (4 °C): 300 *g*, 10 min 2000 *g*, 10 min 10,000 *g*, 30 min 100,000 *g*, 2 h *Washing step* 100,000 *g*, 2 h *Sucrose gradient* 100,000 *g*, 2.5 h	> In Neu1^-/-^ myofibroblasts, there is excessive lysosomal exocytosis and significantly increased secretion of EVs > Neu1^-/-^ myofibroblasts-derived EVs contain molecules of the TGF-β and WNT signaling pathways Several molecules were present on the outer membranes of EVs through glypicans > Neu1^-/-^ myofibroblasts and fibroblast EVs induce proliferation and migration/invasion in WT cells through profibrotic signals > Neu1^-/-^ myofibroblasts EVs convert normal fibroblasts into myofibroblasts through significantly upregulating genes encoding members of the TGF-β and WNT signaling pathways	[78]
**Niemann-Pick Disease type C**	*In vitro* NPC1^-/-^ liver cells from BALB/c mice	Centrifugation: 100,000 *g*, 60 min	> 2-hydroxypropyl-β-cyclodextrin (HPB-CD) treatment stimulates lysosomal exocytosis and EV secretion in a calcium-enhanced manner, which ameliorates endolysosomal cholesterol storage	[83]
	*In vitro* Human NPC1^-/-^ skin fibroblasts and CT43 CHO cells; NPC1 siRNA treated *oligodendroglia* *(Oli-neu)*	Centrifugation: 3000 *g*, 10 min 4000 *g*, 10 min 4000 *g*, 10 min 10,000 *g*, 30 min 100,000 *g*, 60 min *Sucrose gradient* 200,000 *g*, 18 h *Washing step* 100,000 *g*, 1 h	> NPC1^-/-^ fibroblasts secrete significantly higher amounts of EVs; > NPC1^-/-^ CHO cells secrete significantly higher amounts of EVs containing cholesterol; > Cholesterol enhances the release of flotillin-2-positive EVs	[76]
	*In vitro* NPC1^-/-^ fibroblasts NPC1 KO HeLa cells	Centrifugation (4 °C): 2.000 *g*, 30 min 10,000 *g*, 30 min 100,000 *g*, 90 min	> Treatment of NPC1^-/-^cells with LBPA precursor phosphatidylglycerol (PG) reduces endolysosomal cholesterol accumulation through increased secretion in EVs containing cholesterol	[77]
**Niemann-Pick Disease type B**	*In vitro* Human ASMase^-/-^ B lymphocytes	TEM and Flow Cytometry	> LAMP-3/CD63 accumulation, which points to impairment of endocytic trafficking > Microvesicle/lipid particle release may partially bypass the trafficking blockage caused by lipid accumulation > Rapamycin might regulate autophagy/mitophagy and contribute to the clearance of lipid storage by vesicle secretion and lysosomal exocytosis	[84]

Therapeutic stimulation of EV release may have the potential as (pre-)treatment for LSDs through the reduction of LSD specific molecule accumulation inside the cell. This could lower the secondary effects on the endolysosomal pathway by ameliorating the accumulation of MVBs. Advantageous effects of autophagy suppression on the secretion of EVs have been shown in Alzheimer’s Disease and PD^[85,86]^. In these studies, autophagy inhibition by ammonium chloride or bafilomycin A treatment led to the release of EVs containing the accumulated molecules alpha-synuclein and amyloid precursor proteins^[85,86]^. A similar effect was observed in Pompe Disease, where ATG7 was inactivated in the mouse muscles. The autophagy suppression alone led to a 50%-60% reduction of the accumulating glycogen, and after ERT delivery, this was restored to normal levels^[87]^. Unfortunately, the effects on EV secretion were not tested in this study, and it appeared that the accumulating molecule was digested in the cytosol, which improved the cellular phenotype. Contradictory to these results, other studies on Pompe Disease showed improvements in myotubes and muscle tissue after induction of autophagy through TFEB overexpression^[88,89]^. Moreover, induction of autophagy with rapamycin has been shown to lead to higher secretion of lipid particles and CD63+ vesicles in Niemann-Pick type B. This data is in line with studies that predict a way in which autophagy-inducing conditions, mainly rapamycin treatment, have a specific role in unconventional secretion^[90]^. It must be noted that in LSDs, there is no problem with the formation of autophagosomes. Autophagy problems are secondary to dysfunctional lysosomes that create a blockage in the autophagic flux and alter this pathway. Therefore, it must carefully be evaluated to what degree each mutation affects the endolysosomal pathway in all LSDs separately. Alongside the effect of TFEB on autophagy, it can increase lysosomal biogenesis and lysosomal exocytosis. Lysosomal exocytosis could be an alternative route to induce cellular clearance and has been implicated as a mechanism used by MLD and mucolipidosis type I cells^[91,92]^. Lysosomal exocytosis can also be induced by drugs such as 2-hydroxypropyl-β-cyclodextrin (HPB-CD). HPB-CD is used as excipient to facilitate the transport of molecules across membranes as well as treatment to manipulate cellular cholesterol levels. The drug is approved by the FDA and was already used to alleviate cholesterol accumulation in Niemann Pick Type C patients^[93]^. Treatment with HPB-CD can induce calcium-dependent lysosomal exocytosis and EV release^[83]^.

While improving the cellular state sounds promising, the secretion of EVs by LSD cells could have negative effects on neighboring cells or tissues. Their cargo could contribute to the progression of the pathological conditions over time, considering that EVs from LSD cells can contain the accumulated metabolites^[76,94]^. This could especially have a major impact on neuronal tissue and avascular connective tissue. Both tissue types are hard to reach and treat due to the lack of blood supply or barriers, which results in the progression of symptoms after the first line of treatment^[15]^. Increased secretion of the accumulating metabolites could further alter the carefully regulated composition and spatial orientation of the extracellular matrix (ECM). Alterations in the ECM are often seen in LSDs and mostly in MPS, where the primary storage material is a component of the ECM. The specific alterations depend on the type of LSD and may include changes in the size, expression, and arrangement of both fibrous elements and proteoglycans. Furthermore, alterations in the levels of hyaluronic acid (HA) and matrix metalloproteinases (MMPs) are often reported. The ECM modifications in LSDSs can have effects on multiple signaling pathways, cell-matrix and cell-cell interactions^[95]^. The contributions of EVs to these processes were shown by Van de Vlekkert *et al.*, who demonstrated that Neu1^−/−^ myofibroblasts derived-EVs were able to convert normal fibroblasts into myofibroblasts propagating fibrosis^[78]^. This effect was obtained through upregulation of genes in the TGF-β and WNT signaling pathways^[78]^. This suggests that Neu1^−/−^ myofibroblasts derived-EVs can contribute to and/or cause progression of fibrosis. Furthermore, in Krabbe Disease, accumulation of psychosine results in more rigid myelin fluidity and demyelination which occurs through the shedding of myelin by microvesicles^[79]^. This deregulates cell signaling and may introduce focal weak points. In a healthy situation, myelin covers and protects nerve cells to ensure rapid transmission of nerve signals. Consequently, demyelination interrupts signaling of the central and peripheral nervous systems, which contributes to the neurological symptoms seen in Krabbe patients^[96]^. In additional Sphingolipidoses Niemann-Pick type A and C^[97,98]^, changes in the membrane organization and decreased fluidity have been reported. This decrease in membrane fluidity is linked to altered activity of membrane-bound enzymes, carrier-mediated transport, and receptor binding at the cell membrane^[98]^. Whether this influences EVs release in these LSDs remains to be determined. To study EV release in Fabry Disease, Song *et al.* used CRISPR/Cas editing to create GLA-null human embryonic stem cells (hESCs)^[68]^. This model for hypertrophic cardiomyopathy showed impaired autophagic flux and protein turnover combined with increased EV release^[68]^. These studies show the contribution of EVs to the clinical manifestations of LSDs, such as fibrosis, delamination, and hypertrophic cardiomyopathy. More research is needed to investigate the role of the secondary cascade following lysosomal dysfunction in individual LSDs. This will greatly improve our understanding of its pathophysiology inside the cell and its contribution to disease progression. In addition, this could contribute to more targeted and more efficient therapy development. For now, these studies on EVs have demonstrated the potential of vesicles as biomarkers for these symptoms.

## THE POTENTIAL OF EVs AS BIOMARKERS FOR LSDs IN DIAGNOSTICS AND DISEASE MONITORING

EVs are able to reflect the metabolic status of the parent cell through alterations in their cargo, including RNAs, lipids, sugars and proteins^[37]^. They can be isolated from patient material, such as blood, urine, saliva, tear fluid, cerebrospinal fluid, and amniotic liquid^[99-101]^. Moreover, the concentration of EVs in fluids seems to be disease-state dependent as well^[60]^. Therefore, EVs are considered a novel and promising tool in diagnostics and their role as biomarkers has been well studied in multiple diseases with similarities to LSDs, including Alzheimer’s Disease^[102]^ and PD^[103]^. However, their potential as biomarkers in LSDs has yet to be explored.

Since most LSDs are elusive at birth, the diagnostic process of LSDs often relies on suspicion of the physician based on clinical manifestations. However, early diagnosis of LSDs is of utmost importance to start early treatment and prevent irreversible damage to all organ systems involved^[104]^. Current diagnostics of LSDs rely on genetic testing, analyses of undegraded metabolites, and enzymatic activity with dry blood spots (DBSs). Testing on undegraded material is mostly performed on plasma and urine samples; however, this is only available for a limited number of LSDs and lacks specificity. Therefore, confirmation mostly relies on enzymatic testing with DBSs in high-throughput platforms, which is only possible for enzyme deficiencies^[105,106]^. Until now, research on the diagnostic potential of EVs in LSDs is limited. Nevertheless, it has been shown that EVs isolated from Niemann-Pick type C cells contain the accumulated metabolite cholesterol and MLD-EVs contain the accumulated sulfatides^[76,94]^. This might be the case for EVs in other LSDs as well, as the content of EVs reflects the contents and state of the EV-producing cells. Thus, collection of EVs from different types of body fluids could be a valuable source of biomarkers. Their prediction efficiency as biomarkers could be dependent on the location of specific LSD symptoms. For instance, tear fluid has been shown to contain approximately 100-fold higher EV concentrations than plasma^[101]^. Since ocular symptoms such as corneal clouding are often present in LSDs, EVs isolated from tear fluid could be a suitable candidate for biomarker analysis. In other ocular diseases, it has been shown that isolated EVs contain a distinctive protein pattern compared to healthy controls^[107]^. Furthermore, enzyme activity studies can be performed on tear samples as previously shown for MPS I^[108]^. These findings warrant EVs to be studied as a potentially valuable biomarker source in diagnostics, monitoring of disease progression and analysis of treatment efficiency in the future.

As stated before, early indication of LSDs is extremely important to start effective treatment and prevent the development of organ damage. Amniotic fluid could be used for the detection of LSDs. However, most LSDs are typically not evaluated for in the initial standard-of-care workup when babies are born. In several states in the US, parent advocacy has led to the expansion of NBS programs that now include newborn screening in dried blood spots (DBS) for Pompe, Krabbe, Gaucher, Fabry, MPS-I, MPS-II, and Niemann-Pick A/B or a subset^[109]^, while in the Netherlands the screening only includes MPS I^[110]^. Firstly, more LSDs and other metabolic disorders could be included in the standard workup^[111]^. Secondly, when parents choose to have an amniocentesis, co-collection of an amniotic fluid sample could be performed. Isolation of amniotic EVs from these samples could potentially improve early diagnosis. However, as performing an amniocentesis has several risks, collection of EVs from amniotic fluid could be considered alongside scheduled amniocentesis. Keller *et al.* showed that it is possible to separate EVs derived from the fetus and the mother and that these EVs partly represent the renal system of the fetus^[112]^. Even early in development, these EVs could already carry the undegraded material in case of a LSD, as this can normally be detected in urine samples of newborns^[105]^. Using EV-based biomarkers in the diagnostic process may hold the potential to improve the speed of diagnosis and clinical risk assessment of LSDs early in development. In general, the use of EVs could increase the sensitivity of biochemical assays by increasing the concentration of test material. If successful, this could improve both maternal and fetal patient management^[113]^.

Until now, biomarker research in LSDs is limited to the enzyme deficiencies Fabry, Gaucher and MLD. Table 3 shows an overview of all literature related to EVs and their role as biomarkers in these LSDs. EVs could play a role in real-time analysis of disease progression, which could improve personalized treatment protocols. Following this idea, Levstek *et al.* studied urine-derived EVs (uEVs) to evaluate their potential in the prediction of nephropathy progression in Fabry Disease^[114]^. In almost all Fabry patients， the kidneys are affected. In this study, it was shown that uEVs from Fabry patients with nephropathy contained increased expression of miR-29a-3p and miR-200a-3p. On the other hand, patients who were not affected by renal dysfunction had higher expression levels of miR-30b-5p, which plays a protective role in podocyte injury. This shows that screening of uEVs in these patients could help define the presence and status of nephropathy^[114]^. For patients with some residual enzyme activity, this method could be used to assess when to start ERT, while for patients with little or no residual enzyme activity, this could mark the start of dialysis^[115]^. Gaucher Disease includes a major genetic risk factor for the development of PD in these patients. Tatiana *et al.* performed proteomic profiling of EV proteins from blood plasma of Gaucher patients to find any association with PD^[116]^. While they showed increased EV size in patient material, proteomics did not reveal any PD-associated proteins^[116]^. Additional EV cargo, such as lipids, sugars and RNAs, were not tested in this study.

**Table 3:** Extracellular vesicles as biomarkers for LSDs

**LSD**	**Study design**	**Patient material**	**EV cargo (biomarker)**	**Isolation method**	**Major results**	**Reference**
**Fabry Disease**	Patient material	Urine	*Negative:* miR-29a-3p and miR-200a-3p *Positive:*miR-30b-5p	Centrifugation: 2000 *g*, 15 min Storage -80 °C Mix urine with PBS and EDTA AMICON Ultra-15 Spin-filter 4000 *g*, 10 min SEC (70 nm column) AMICON Ultra-4 Spin-Filter 4000 *g*, 6 min at 4 °C	> uEVs contain increased expression of miR-29a-3p and miR-200a-3p over time in patients with nephrology > Over 10 years, uEVs of patients without renal dysfunction contained increased levels of miR-30b-5p, which has a protective role in podocyte injury	[114]
	Patient material *In vitro* HUVEC	Plasma	miR-126-3p	*Plasma EVs* Centrifugation: 1500 *g*, 10 min 3000 *g*, 30 min 10,000 *g*, 30 min 100,000 g, 1 h 45 min at 4 °C *HUVEC EVs* Centrifugation: 300 *g*, 10 min 2000 *g*, 20 min 10,000 *g*, 30 min 110,000 *g*, 70 min *Washing step* 110,000 *g*, 70 min	> Circulating miR-126 levels physiologically increase with age > sEVs of Fabry Disease patients contain higher miR-126-3p levels > Glycosphingolipid accumulation induces premature senescence in HUVECs and increases miR-126-3p levels > Fabry Disease may aggravate the normal aging process	[117]
**MLD**	*In vivo* MLD mice (ARSA^-/-^)	H*In vivo* emi sagittal brains		Blade dissociation 100 μm mesh filtration 40 μm mesh filtration Centrifugation: 300 *g*, 10 min 2000 *g*, 20 min 10,000 *g*, 30 min 10,000 *g*, 30 min 100,000 *g*, 90 min *Sucrose gradient* 200,000 *g*, 16 h 100,000 *g*, 90 min	> Brain derived-EV concentration increased in parallel with age > MLD brain-EVs are significantly larger at 3 months compared to day 30 or 6 months > There is loading of accumulating sulfatides into MLD-EVs and alterations in the loading of different ceramides > Progressive decrease of FAs and FAHFAs in MLD-EVs	[94]
**Gaucher Disease**	Patient material	Plasma	Increased size	Centrifugation: 2000 *g*, 30 min 16.000 *g*, 30 min 110,000 *g*, 2 h *Washing step* Gentle swaying for 1h at 4 °C Filtered (0.45 μm)	> EVs of Gaucher Disease patients are increased in size and have altered morphology > Proteomic profiling of exosomal proteins did not reveal any proteins associated with PD pathogenesis	[116]
	Patient material	Serum	Increased number of MPs	Centrifugation (20 °C): 1550 *g*, 20 min 18.800 *g*, 30 min *Washing step* 18.800 *g*, 30 min	> Higher levels of MPs from different blood cells in patient samples both before and after ERT compared to controls > After 1 year of ERT, the total MPs in GD were significantly decreased	[118]

FAs: Fatty acids; FAHFAs: fatty acid ester of hydroxyl fatty acids; MPs: microparticles; SEC: size exclusion chromatography.

In addition to protein and miRNA content, the lipid composition of EVs could be involved in the reflection of disease pathology. Hence, Pergande *et al.* performed an untargeted lipidomic analysis of brain-derived EVs isolated from MLD mice^[94]^. In the sphingolipidosis MLD, deficiency of the aryl-sulfatase A (ARSA) enzyme leads to accumulation of sulfatides, including multiple sulfated glycolipids^[94]^. The accumulation of sulfatides causes severe neurological symptoms in MLD patients. Sulfatides have been found in EVs before, for instance, in EVs isolated from plasma and oligodendrocytes^[119,120]^. Pergande *et al.* showed that MLD-EVs contained numerous altered lipid species from a broad range of lipid classes^[94]^. This included sulfatides and their ceramide precursors as well as fatty acids (FAs), fatty acid esters of hydroxyl fatty acids (FAHFAs) and acylglycerol lipids. The sulfatides include mostly 18-carbon fatty acid chains, which predominantly accumulate in MLD mice. Furthermore, these lipid alterations in EVs were found to be age-dependent and are proposed as relevant candidates for MLD biomarkers^[94]^.

Further research into specific LSD-related biomarkers could improve our understanding of disease pathology as well as improve patient care. Moreover, unraveling the tissue origin of EVs secreted in different body fluids through specific tissue biomarkers could improve our understanding of disease location and progression and thus improve treatment. We suspect that the use of algorithms in EV research will progress in the upcoming years and will be of great value to analyzing EV data from body fluids in relation to disease^[51,121-123]^.

## IMPROVEMENT OF THERAPEUTIC DELIVERY WITH EXTRACELLULAR VESICLES AS DRUG DELIVERY VEHICLES

EVs are biocompatible and have intrinsic tissue-penetrating abilities^[124]^. In addition, EVs show low immunogenicity and are stable in circulation due to their negatively charged surface and their ability to avoid the mononuclear phagocytic system upon modification^[62]^. These features qualify EVs as potential new therapeutic drug delivery vehicles. The ability of EVs to cross the BBB could open doors to the development of novel treatment and delivery strategies for LSDs with neurological deficits^[38]^. Furthermore, it makes EVs a more desirable delivery tool over existing nanotechnologies such as liposomes, synthetic polymers and gold nanoparticles^[125,126]^. EVs naturally contain lysosomal components including heparan-alpha-glucosaminide N-acetyltransferase (HGSNAT)^[50]^, beta-glucocerebrosidase (GBA), N-acetylgalactosamine-6-sulfate sulfatase (GALNS)^[127]^, cystinosis (CTNS)^[128]^, deficient in MPS III, Gaucher, MPS IVA, and Cystinosin respectively. Considering the interplay between the endolysosomal pathway and EV secretion, there could be a broader range of natively loaded LSD deficient proteins into EVs^[128]^, making them an interesting vehicle for ERT.

EV uptake into recipient cells can take place through a variety of different pathways. EV internalization predominantly occurs through endocytosis, which includes macropinocytosis, receptor-mediated, clathrin-dependent and clathrin-independent mechanisms^[129]^. In addition, EVs could also enter the cells through phagocytosis. Additionally, EVs may also exert phenotypical effects on target cells without entry through direct receptor-ligand interactions^[124,130]^. Most of the internalization pathways lead EVs to the endolysosomal pathway. Numerous studies have shown strong colocalization of EVs with lysosomes, reaching up to ~50%-60% in fibroblasts^[130]^. For most therapies, this will be a disadvantage when (intraluminal) EV cargo needs to be delivered to the cytosol of nucleus. In that case, EVs need to escape the endosome to avoid degradation in the lysosome. It is largely unknown how EVs can naturally induce endosomal escape; however, there is some evidence that fusion between the membranes of EVs and the late endosomal membranes plays a role^[124]^, potentially under the influence of acidification in the endolysosomal environment^[131]^. Nevertheless, EVs being trafficked towards the lysosome could be of substantial benefit for the delivery of therapeutics in the treatment of LSDs. However, whether the same distribution pattern towards the dysfunctional lysosomes in LSD cells will be seen remains to be determined.

Already a decade ago, Coulson-Thomas *et al.* showed that transplantation of human umbilical mesenchymal stem cells improves the corneal defects of MPS-7 mice^[132]^. After injections of these MSCs, improvements were hypothesized to be mediated through MSC-derived EVs^[132]^. At the time, EVs were gaining more recognition for their potential therapeutic abilities^[133]^. To date, several studies have shown that overexpression of lysosomal enzymes in cells leads to passive loading into EVs, including the deficient enzymes of Fabry^[128]^, MPS IIIA^[128]^, MPS IVA^[127]^, and Batten Disease^[134]^. An overview of all studies regarding EVs in the treatment of LSDs can be found in Table 4.

**Table 4:** Extracellular vesicles as therapeutic carriers for LSD treatment

**LSD**	**Study design**	**EV cargo**	**EV concentration**	**Isolation method**	**Major results**	**Reference**
**Fabry Disease**	EVs from stable GLA expressing CHO DG44 cells *In vitro* HEK293T and NAEC cells *In vivo* GLA^-/-^ mice	GLA	*In vitro* 2.5 μg EVs/ml (c.a. 125 ng GLA/ml) *In vivo* 1 mg/kg GLA	Centrifugation (4 °C): 3900 rpm, 15 min (free enzyme) 300 *g*, 10 min 2000 *g*, 10 min 10,000 *g*, 20 min VIVAspin 30,000 KDa 7.000 *g*, 10 min Precipitation o/n with ‘Total Exosome Isolation Reagent’ 16.000 *g*, 1 h	> Significant amounts of GLA in EV lysates compared to whole cell lysates > Predominant contribution of clathrin-mediated endocytosis and macropynocytosis in EV uptake > EVs protect GLA and increase its enzymatic activity upon delivery compared to commercial GLA and agalsidase alfa used in ERT > EVs were well tolerated *in vivo *and distributed among all main organs, including the brain after i.a. administration	[128]
**MPS IIIA**	Transient SGSH expressing HEK293 cells *In vitro* HEK293T and NAEC cells	SGSH	2.5 μg EVs/ml	Centrifugation (4 °C): 3900 rpm, 15 min (free enzyme) 300 *g*, 10 min 2000 *g*, 10 min 10,000 *g*, 20 min VIVAspin 30,000 KDa 7000 *g*, 10 min Precipitation o/n with ‘Total Exosome Isolation Reagent’ 16.000 g, 1 h	> Significant amounts of SGSH in EV lysates compared to whole cell lysates > EVs restore lysosomal function much more efficiently than the recombinant enzyme in clinical use > *In vivo*, EVs were well tolerated and distributed among all main organs, including the brain	[128]
**MPS IVA**	hUMSC-derived EVs *In vitro* MPS IVA deficient patient fibroblasts	GALNS	Co-cultures	*Conditioned medium* 3000 rpm, 15-20 min at 4 °C Centrifugation: 300 *g*, 10 min 10,000 *g*, 30 min 0.22-μm filter 100,000 *g*, 90 min	> UMSC-EVs containing functional GALNS enzyme and can deliver it to CALNS deficient cells; > Mannose-6-phosphate inhibition did not affect enzyme delivery after EV treatment; > EVs are a function and novel technique for reducing GAG accumulation in cells of avascular tissues	[127]
**Cystinosis**	amMSCs and bmMSCs *In vitro* Patient derived CTNS^-/- ^skin fibroblast and ciPTEC	CTNS	Co-cultures, 1-2 mg microvesicles/mg fibroblast protein	Centrifugation (4 °C): 300 *g*, 5 min 100,000 *g*, 2 h *Washing* 100,000 *g*, 1 h *Washing* 100,000 *g*, 1 h	> Amniotic fluid or bone marrow derived hMSCs, reduce pathologic cystine accumulation in co-culture with patient CTNS-^/-^ fibroblasts or proximal tubular cells; > MSC derived EVs contain wildtype cystinosin protein and CTNS mRNA	[135]
	EVs derived from Spodoptera frugiperda (Sf9) cells infected with Baculovirus *In vitro* CTNS^-/- ^fibroblast	CTNS	-	0.2 μm filter Dialysis to dilution of 1/2500, or 2 (cut-off 3.5 kDa) Centrifugation: 140,000 *g*, 3 h followed filter-sterilization	> Vesicle addition to CTNS^-/- ^fibroblast leads to 57% cystine depletion > LC-MS/MS analysis of the vesicles shows three cystinosin peptides with 7.4% coverage of the human cystinosin sequence > Quantitative tandem mass spectrometry showed 0.1 and 1.2 pmol cystinosin per mg of vesicle protein > Both western blot and RT-PCR confirmed the presence of cystinosin in these vesicles	[136]
	EVs derived from Spodoptera frugiperda (Sf9) cells infected with Baculovirus containing the hCTNS-GFP *In vitro* CTNS^-/- ^fibroblast Rabbit globes	CTNS	Gas-permeable stoppers containing 50 ml medium with or without the 10^11^/ml EVs	Freeze-thaw snap freezing medium Thawed Dialyzed into Ham’s F12, 48 h at 1:2500 dilution 0.22 μ filter Stored at 4 °C	> Baculovirus infected Spodoptera frugiperda cells (Sf9) spontaneously produce EVs that contain cystinosin > EVs are detected in CTNS^-/-^ fibroblast with perinuclear and cytoplasmic distribution after treatment; > Upon ex vivo ocular EV delivery, GFP signal is detected for 48 h	[137]
**CLN2**	TPPI-plasmid DNA transfected peritoneal macrophages or sonication and permeabilization of EVs with saponin to load TPPI ***In vitro*** CLN2^-/-^ skin fibroblasts *In vivo* Intraperitoneal EV administration in late-infantile neuronal ceroid lipofuscinosis (LINCL) mice	TPPI	*In vitro* 10^10^/mL *In vivo* Intraperitoneal administration Bioimaging: 2 × 10^11^ in 200 μL saline Brain accumulation: 1 × 10^10^ in 100 μL Therapeutic efficiency: 4.3 × 10^12^ in 150 μL	Centrifugation: 300 *g*, 10 min 1000 *g*, 20 min 10,000 *g*, 30 min 0.2 μm syringe filter 100,000 *g*, 4 h *Washing* 120,000 *g*, 70 min *Washing* 120,000 *g*, 70 min Sonification or permeabilization	> Both transfection with TPPI plasmid and sonification/permeabilization lead to proficient incorporation of functional TPP1 into macrophage-EVs (EV-TPP1); > EVs significantly increase stability of TPP1 against protease degradation and provide efficient TPP1 delivery to CLN2^-/- ^fibroblasts; > Around 70% of the EV-TPP1 is delivered to lysosomes; > Intraperitoneal administration of EV-TPP1 leads to accumulation in the brain of LINCL mice, which increased lifespan	[134]
	EVs derived from mice bone marrow-derived macrophages *In vitro* CLN2^-/-^ primary cortical neurons *In vivo* Intrathecal and intranasal routes CLN2^-/-^ LINCL mice	TPPI	*In vitro* 5 × 10^11^ EVs *In vivo* i.v. 6 × 10^11^ in 200 µL i.p. 6 × 10^11^ in 200 µL i.t. 1.5 × 10^11^ in 50 µL i.n. 6 × 10^11^ in 20 µL	Centrifugation: 300 *g*, 10 min 1000 *g*, 20 min 10,000 *g*, 30 min 0.2 μm syringe filter 100,000 *g*, 4 h Washing 120,000 *g*, 70 min Washing 120,000 *g*, 70 min Sonification	> EV-TPP1 treatment though i.t. and i.n. routes lead to accumulation in the brain, decreased neuroinflammation and neurodegeneration and reduced aggregation in CLN2^-/-^ mice; > EV-TPP1 treatment though i.v. and i.p, administration leads delivery to the liver, spleen, kidney, and lungs; > Combined i.t. and i.p. injection significantly prolonged lifespan in CLN2^-/-^ mice	[138]

ciPTEC: Proximal tubular epithelial cells; CTNS: cystinosis; GALNS: acetylgalactosamine-6-sulfate sulfatase; GLA: alpha‐galactosidase A; i.n.: intranasal; i.p.: intraperitoneal; i.t.: intrathecal; i.v.: intravenous; SGSH: N‐sulfoglucosamine sulfohydrolase; TPPI: tripeptidyl peptidase-1; UMSC: umbilical mesenchymal stem cells.

While EVs improve drug stability and circulation time^[139]^, they can be further optimized to improve EV uptake and biodistribution and obtain targeted delivery. All cells release EVs, but the number, composition, functionality and physicochemical characteristics vary between cell sources^[62]^. For instance, MSC-derived EVs have immunomodulatory and anti-inflammatory effects. Recently, unmodified MSC-EVs were used to treat osteoarthritis and resulted in amelioration of disease progression through induced polarization of M2-type macrophages among other effects^[140]^. These positive effects on disease related to connective tissue could be beneficial to treat residual disease and target the connective tissue in LSD patients. Furthermore, the native characteristics of MSC-EVs could be further exploited through engineering. This might lead to delivery of the loaded protein while also promoting tissue repair and regeneration within the same treatment. Moreover, EV functionality is dependent on the target as well, considering that some EVs are better taken up by one cell type compared to another^[36]^. Therefore, therapy specific selection of the optimal cell source, cell cultivation procedure, EV extraction and purification methods, and storage might be needed.

Recently, Seras-Franzoso *et al.* showed that stabile GLA expressing cell lines were able to produce EVs that protected the enzymes from proteases, and subsequently improved enzymatic delivery directly compared to soluble GLA and clinically approved ERT agalsidase alfa^[128]^. In addition, analysis of the GLA biodistribution showed that EVs were able to deliver significantly more active enzymes to the kidneys than non‐encapsulated enzymes. It must be noted that agalsidase alfa used in ERT is governed by the presence of the mannose-6-receptor to increase uptake. This receptor is highly expressed in the liver and therefore might interfere with the biodistribution of non-encapsulated enzymes ^[141]^. Furthermore, they showed the uptake of fluorescent labelled EV-GLA in the brain parenchyma with confocal microscopy an hour post-intra‐arterially (i.a.) administration through cannulation of the external carotid artery. This delivery across the BBB could not be detected after intravenous administration through the tail vein^[128]^. Likewise, Haney *et al.* showed the loading of tripeptidyl peptidase-1 (TPP1) into EVs, which greatly improved enzyme stability^[134,138]^. After showing functional delivery of the TPP1 in EV treated fibroblast, the authors examined intraperitoneal administration of EVs in late-infantile neuronal ceroid lipofuscinosis (LINCL) mice to study EV distribution towards the brain. This type of administration led to accumulation of the enzyme in the brain. However, a clear distribution towards peripheral organs was also seen, including the liver, spleen, kidneys and lungs^[134]^. Therefore, they examined different EV administration routes in mice in another study to see the effect on crossing the BBB. Both intranasal and intrathecal administration resulted in accumulation of TPP1 in the brain and spinal cord, which reduced aggregation of lysosomal storage material, neurodegeneration and neuroinflammation. Importantly, these administration routes showed lower distribution of the EVs towards the peripheral organs compared to intraperitoneal and intravenous administration^[138]^. The loading of enzymes into EVs and their distribution towards the central nervous system (CNS) shows the potential of EVs as therapeutic carriers to treat the CNS. Thus, EVs could be used to load and protect ERT to improve biodistribution and lower antibody generation in patients.

## ENGINEERING OF EXTRACELLULAR VESICLES FOR THERAPEUTIC TREATMENT OF LSDs

While native EVs have multiple features that qualify them as promising drug delivery vehicle, endogenous cargo loading, circulation time and tissue targeting are often inadequate. Biodistribution studies often show accumulation of EVs in the liver, spleen, gastrointestinal tract and lungs. However, the specific pattern of distribution seems to depend on the cell type the EVs are derived from, the route of administration^[142]^ as well as the injected dose^[64]^. Several studies have demonstrated a role for integrins in tissue targeting^[143,144]^. Integrins are enriched in EVs through their association with tetraspanins^[145]^. For instance, integrin expression patterns in tumor-derived EVs play a role in the uptake of EVs by organ-specific cells and creation of a pre-metastatic niche^[143]^. The use of fluorescent labelling and tracking techniques are valuable tools to study and optimize the distribution pattern of (engineered) EVs. For an overview of the currently used labelling and tracking techniques in the EV field, we refer to the review of Kooijmans *et al.*^[129]^*. *To further optimize performance, EVs can be modified and engineered. To date, studies on EV engineering for LSD treatment have solely focused on actively increasing therapeutic cargo loading. These modifications can be obtained through a variety of different exogenous and endogenous methods. In exogenous techniques, EVs are therapeutically loaded post-isolation by simple incubation, electroporation, sonication, extrusion, and freeze-thawing. These techniques have variable degrees of success and can lead to EV or cargo aggregation^[146,147]^. Uniquely, EVs can also be endogenously altered by biologically engineering the cells that produce them. This strategy can equip EVs with new moieties while preserving their membrane integrity, which is an advantage over exogenous loading techniques^[123]^.

Therapeutic protein loading into EVs is often achieved through parental cell engineering with genes that encode for the therapeutic protein fused to a membrane protein enriched in EVs or a transmembrane domain. These loading strategies may include the use of EV enriched proteins such as tetraspanins (CD9, CD63 and CD81), PTGFRN, and BASP1^[148]^ or transmembrane domains such as the N-terminal fragment of syntenin^[149]^. With this strategy, the therapeutic protein will naturally be incorporated into EVs during biogenesis. This approach allows for both internal and external display of the therapeutic protein^[150]^. Successful protein loading and delivery, both *in vitro* and *in vivo*, has been shown in multiple studies^[150-152]^. This shows the potential for active enzyme loading and accumulation in EVs to increase functional delivery. When designing active enzyme loading strategies, it must be considered that lysosomal enzymes often require post-translational modification. These modifications need to take place before loading enzymes into EVs to eventually lead to functional delivery upon EV treatment^[153,154]^.

To date, the only LSD for which active enzyme loading into EVs has been studied is Gaucher Disease. An overview of these studies can be found in Table 5. In patients with Gaucher Disease, the GBA enzyme is deficient, which leads to symptoms that include neurological complications. Do *et al.* showed active GBA loading into EVs with the use of vesicular stomatitis virus glycoprotein (VSV-G)^[153]^. In addition to loading and potential uptake through the VLDL receptor on the surface of recipient cells, VSV-G can also induce endosomal escape^[90]^. It should be noted that induced endosomal escape could potentially counteract the goal of this EV treatment by enabling the EV cargo escape their route to the lysosome. Despite that, Do *et al.* show significant colocalization of the EVs throughout uptake with the endosome, late-endosome and lysosome in an *in vitro *HEK293 cell model^[153]^. However, it is possible that delivery of GBA to these cellular structures would even further increase with the use of a loading strategy that does not additionally induce endosomal escape. Flow cytometry analysis showed a 73%-75% uptake rate based on mean fluorescent intensity of the co-loaded GFP^[153]^. Although this study focused on *in vitro* cell culture models, it underlines the potential of active loading strategies into EVs to deliver deficient enzymes as next-generation ERT. In the future, such therapies may lower antibody generation to ERT in patients and deliver functional enzymes to target organs including the central nervous system (CNS), which is severely affected in multiple LSDs.

**Table 5:** Engineered extracellular vesicles to treat LSDs

**LSD**	**Study design**	**EV cargo**	**Readout**	**EV concentration**		**Isolation method**	**Major results**	**Reference**
**Gaucher Disease**	EVs from HEK293T cells *Active loading* *VSVG-GFP-GBA*	GBA	*In vitro* HEK293T, U7 and HepG2		0.3 µg/μl	Centrifugation: 1500 *g*, 10 min 0.2 µm syringe filter Mixed with ExoQuick-TC solution (1:4) o/n at 4 °C 3000 *g*, 90 min at 4 °C	> HEK cells transfected with VSVG-GFP-GBA constructs secrete EVs that contain significantly more active GBA compared to control EVs; > The uptake of green, fluorescent dye labelled GBA-EVs was 73%-75%; > Upon treatment, GBA loaded EVs co-localize with lysosomes and lead to the delivery of functional GBA (40%-45%)	[153]
**CLN2**	TPPI-plasmid DNA transfected peritoneal macrophages or sonication and permeabilization of EVs with saponin to load TPPI *In vitro* CLN2^-/-^ skin fibroblasts *In vivo* Intraperitoneal EV administration in late-infantile neuronal ceroid lipofuscinosis (LINCL) mice	TPPI	*In vitro* IC21 cells, neuronal PC12 cells and Human skin CLN2^-/-^ fibroblasts *In vivo* LICL mice		*In vitro* 10^10^/mL *In vivo* Intraperitoneal administration Bioimaging: 2 × 10^11^ in 200 μL saline Brain accumulation: 1 × 10^10^ in 100 μL Therapeutic efficiency: 4.3 × 10^12^ in 150 μL	Centrifugation: 300 *g*, 10 min 1000 *g*, 20 min 10,000 *g*, 30 min 0.2 μm syringe filter 100,000 *g*, 4 h *Washing* 120.*000 g*, 70 min *Washing* 120,000 *g*, 70 min Sonification or permeabilization	> Both transfection with TPPI plasmid and sonification/permeabilization lead to proficient incorporation of functional TPP1 into macrophage-EVs (EV-TPP1) > EVs significantly increase stability of TPP1 against protease degradation and provide efficient TPP1 delivery to CLN2^-/- ^fibroblasts > Around 70% of the EV-TPP1 is delivered to lysosomes > Intraperitoneal administration of EV-TPP1 leads to accumulation in the brain of LINCL mice, which increased lifespan	[134]
	EVs derived from mice bone marrow-derived macrophages *In vitro* CLN2^-/-^ primary cortical neurons *In vivo* Intrathecal and intranasal routes CLN2^-/-^ LINCL mice	TPPI	*In vitro* Primary bone murine marrow-derived macrophages, cortical neurons and IC21 cells *In vivo* LICL mice		*In vitro* 5 × 10^11^ EVs *In vivo* i.v. 6 × 10^11^ in 200 µL i.p. 6 × 10^11^ in 200 µL i.t. 1.5 × 10^11^ in 50 µL i.n. 6 × 10^11^ in 20 µL	Centrifugation: 300 *g*, 10 min 1000 *g*, 20 min 10,000 *g*, 30 min 0.2 μm syringe filter 100,000 *g*, 4 h Washing 120,000 *g*, 70 min Washing 120,000 *g*, 70 min Sonification	> EV-TPP1 treatment though i.t. and i.n. routes lead to accumulation in the brain, decreased neuroinflammation and neurodegeneration and reduced aggregation in CLN2^-/-^ mice > EV-TPP1 treatment though i.v. and i.p, administration leads delivery to the liver, spleen, kidney, and lungs. > Combined i.t. and i.p. injection significantly prolonged lifespan in CLN2^-/-^ mice	[138]

GBA: β-Glucocerebrosidase/β-glucosidase; GCase: Glucocerebrosidase; VSV-G: Vesicular stomatitis virus G.

## THE USE OF EVs IN FUTURE THERAPEUTIC TREATMENT OF LSDs

Engineered EVs have already been explored for multiple purposes, including regenerative therapy^[155]^, immune modulation^[156]^, delivery of small molecular drugs^[157]^, vaccines^[158]^, therapeutic proteins, and nucleic acids^[159]^. For instance, cardiac progenitor cell-derived EVs loaded with miR-322 outperformed their unloaded controls in enhancing angiogenesis in mice after ischemic injury^[155]^. Furthermore, numerous small molecular drugs have been successfully incorporated into EVs, including curcumin. The loading of curcumin into EVs enhanced its delivery and anti-inflammatory activity^[156]^. Drug loading into EVs has been shown to improve accumulation in target cells and enhance drug stability and circulation time^[139]^. Currently, multiple groups are exploring the potential of employing EV engineering for the development of therapeutic treatments. The variety and possibilities of this technique could greatly improve therapeutic options for LSDs in the future.

Alongside the mentioned positive effects of using EVs to deliver ERT, delivery of other forms of LSD treatment might also benefit from this camouflage strategy. This may include SRT and PCT, which use small drugs to inhibit the production of the accumulating substrate and stabilize native structures of mutated enzymes, respectively^[26,27]^. Loading both SRT and CPT into EVs might improve their distribution and potential delivery to the CNS. Furthermore, newly discovered molecular drugs might benefit from being loaded into EVs as well by increased loading efficiency, protection, improved circulation and crossing of biological membranes. As discussed before, in most LSDs, autophagy is altered and affected cells might reduce their accumulation through secretion in the hope of maintaining homeostasis. Curcumin treatment has been shown to alleviate the phenotype of Niemann-Pick type C through the elevation of cytosolic calcium levels^[160]^. Additionally, it has been shown that curcumin promotes EV section in cells that represent Niemann-Pick type C, inducing cholesterol shuttling out of the cell^[161]^. Loading and delivery of curcumin via EVs may be a therapeutic approach to treat Niemann-Pick type C.

In addition to enzyme deficiencies, there are other subgroups of LSDs that include deficiencies in integral membrane proteins, transporters and proteins involved in the post-translational modification, trafficking, or regulation. For these subgroups, there are limited treatment options available. Gene therapy was evaluated to treat Niemann-Pick type C in which patients are missing or have defective NPC1 transporters. Currently, Evox Therapeutics filed a patent to facilitate the loading of NPC1 fused to syntenin/CD63 into EVs (Patent number: WO2019/092287 AI). The use of EVs to transport NPC1 will stabilize the integral membrane protein through the presence of lipid molecules^[162]^. This strategy of EV engineering could potentially be a step forward in designing treatment strategies for currently untreatable LSDs.

Current challenges of EVs that must be addressed to improve their therapeutic efficacy include their rapid clearance and limited targeted delivery. Possible solutions may involve adaptation of administration routes depending on the target tissue. Local administration might be preferred for connective tissues with limited blood supply, whereas intranasal or intrathecal administration might be beneficial to target the CNS^[138]^. Moreover, modification of the EV surface may improve EV circulation and tissue targeting^[63,163,164]^. Kamerkar *et al.* have reported that the presence of CD47 on EV surface inhibited EV uptake and clearance by macrophages and monocytes, which may lead to increased cargo delivery^[165]^. However, considering that LSDs are monogenetic disorders, the monocyte-macrophage system is affected in multiple LSDs as well^[166]^. Therefore, the uptake of drug-loaded EVs by these immune cells could also be beneficial. Several bioengineering strategies have been developed to increase the affinity of EVs for specific recipient cells. These approaches include receptor-ligand, enzymatic, and antigen-antibody or a combination of these methods. For instance, EVs containing a LAMP2b-designed ankyrin repeat protein (DARPin) G3 fusion protein are able to specifically target HER-2 positive breast cancers through receptor-ligand interaction. DARPins are a class of synthetic peptides that can bind biological receptors with high specificity and binding affinity and specificity^[167]^. An adaptation of the EV membrane that both improved circulation time and cell specificity was shown by the addition of epidermal growth factor receptor (EGFR) nanobodies conjugated to phospholipid (DMPE)-polyethylene glycol (PEG). The shielding properties of PEG compromised cell binding and increased detectability of EVs in the plasma from less than 10 min to over 60 min post-injection. At the same time, the EGFR nanobodies directed the EVs towards tumor cells overexpressing the EGFR^[63]^. Surface modification can also be used to evade phagocytosis by the mononuclear phagocyte system (MPS). These strategies will increase circulation time which will improve the chance of EV uptake by the specific recipient cells and can be used to increase EV accumulation and cargo delivery in targeted tissues^[63]^. Alongside biological engineering, EVs can externally be altered through chemical or synthetic engineering by using click chemistry or fusion with nanotechnologies^[62,129]^. All the different engineering strategies make it possible to optimize EVs to potentially target any tissue and disease.

## EV-MEDIATED GENE EDITING: THE POTENTIAL ROUTE TO A PERMANENT CURE FOR LSDs

LSDs are genetic disorders and recent studies have been exploring the possibilities of gene editing to correct their mutations^[168]^. CRISPR/Cas technology possesses the ability to selectively target genes and create deletions, insertions, and base pair substitutions. In this technology, the Cas9 protein is guided towards a sequence with the help of single guide RNA (sgRNA). When using the native Cas9 protein, this results in a specific double‐stranded break (DSB) in the targeted genomic DNA that can be corrected by the native DNA repair mechanisms of the cell^[169]^. The repair of DSBs is associated with non-specific mutations, since it is typically repaired by the error-prone non-homologous end-joining (NHEJ) pathway, resulting in point mutations, deletions, and frameshifts^[170]^. To create precise changes in the DNA, the homology-directed repair (HDR) pathway utilizes a DNA repair template which shows homology in the sequences surrounding the DSB. This template can be delivered as a double-stranded DNA template or a single-stranded DNA oligo, which can contain a desired restorative mutation. However, HDR occurs at a relatively low frequency as compared to NHEJ. Thus, in order to generate base pair substitutions without the presence of NHEJ-mediated non-specific mutations, Base Editing (BE) was developed. Here, enzymatic groups that are able to convert specific nucleotides are attached to dCas9, an adapted form of Cas9 whose endonuclease activity is removed through point mutations in its endonuclease domains. As a result, Cas9 will no longer create DSBs while still interacting with specific genomic sequences through the sgRNAs, and the base editing enzymatic groups are able to generate nucleotide substitutions in the sgRNA target sequence. There are different types of base editors, including cytosine base editors (CBEs) and adenine base editors (ABEs) that can mediate single base changes in the genome. Base editors can induce these alterations without requiring DSBs, HDR processes, or donor DNA templates^[171-173]^. The main drawback of these techniques is that the type of substitutions that can be made are currently limited, and since these substitutions could be made throughout the ~20 nt target sequence of the sgRNA, only a limited number of target sequences are suitable for base editing techniques.

Recently, Anzalone *et al.* reported a new versatile and precise genome editing method called prime editing^[174]^. Prime editing does not require a separate donor DNA repair template to precisely edit the DNA. Instead, the needed repair template is incorporated into the sgRNA combined with the desired edit, now called prime editing guide RNA (pegRNA). Furthermore, a catalytically impaired Cas9 nickase is fused to an engineered reverse transcriptase, now called the prime editor (PE). As prime editing is based on the use of a Cas9 nickase, which only cuts one strand of the double helix, DSBs and the subsequent error-prone NHEJ repair process are avoided. After the pegRNA guids the PE towards the target sequence, the reverse transcriptase domain of the PE uses the repair sequence added to the pegRNA to template reverse transcription of the desired edit. This leads to direct transcription of the DNA onto the nicked target DNA strand. The edited DNA strand will replace the original DNA strand, which creates a heteroduplex of one edited strand and one unedited strand. The edited strand will be favored due to the inherent preference of the endogenous endonuclease FEN1 to excise 5’ flaps. This leads to hybridization of the edited 3’ flap being favored copying the edit onto the unedited strand, completing the process. Prime editing can incorporate all the potential 12 modifications; accordingly, prime editing could, in principle, correct up to 89% of known genetic variants associated with human diseases^[174,175]^. In addition to efficient gene correction in cell lines, prime editing has already been shown to be as efficient in 3D grown organoids derived from patients with inherited metabolic disorders^[176]^. This substantially expands the scope and capabilities of genome editing, but the *in vivo* delivery remains challenging^[174]^.

The CRISPR/Cas system can be delivered either as plasmid DNA, mRNA or as ribonucleoprotein (RNP) complex. Delivery of the RNP complex has advantages over plasmid and mRNA delivery, including lower off-target effects, and faster and more efficient gene editing^[177]^. Nevertheless, there are limitations to *in vivo* RNP complex delivery, including its large size, negative charge, immunogenicity, pre-existing antibodies^[178]^, and rapid degradation^[179]^. Loading of the RNP complex into EVs could potentially overcome most of its limitations, as well as lowering its off-target effects by increased tissue targeting.

Whereas EV trafficking towards the lysosome after uptake by recipient cells is an advantage when using engineered EVs to deliver ERT, for the functional delivery of the RNP complex, endosomal escape is required, as the RNP complex needs to be able to reach the cell nucleus to induce genomic editing. Incorporation of viral fusogenic proteins into the EV membrane could increase the relatively low efficiency of endosomal escape of unaltered EVs. The viral protein VSV-G is known to incorporate into the EV membrane upon transfection of the cells with VSV-G encoding plasmids^[180]^. This protein specifically induces endosomal escape, as it requires the decrease in pH in the late endosome to undergo the conformational transformation that induces the membrane fusion that facilitates cargo release. This will greatly increase endosomal escape and with that the delivery of the EV loaded RNP complexes or other therapeutic components that require delivery to the cytoplasm. Nevertheless, the use of viral proteins can be problematic due to their cytotoxicity and potential immunogenicity^[181]^. The substitution of these viral proteins is a hurdle that needs to be overcome. EVs may contain some endogenous capacity to escape the endosome that could be explored. For instance, through the incorporation of endogenous proteins with fusogenic activity like syncytins. Syncytins are incorporated into placenta-derived EVs and mediate uptake by recipient cells^[182]^. Their incorporation into the membrane may facilitate EV fusion and subsequently deliver the EV cargo^[129,183]^. It is hypothesized that EVs contain additional proteins that facilitate endosomal escape through membrane fusion, as treatment of unmodified EVs with proteinases has been shown to abrogate their capability of membrane fusion^[131]^. The use of endogenous proteins with fusogenic activity to induce endosomal escape for the delivery of CRISPR/Cas could increase the safety of these EVs.

**Figure 2:**
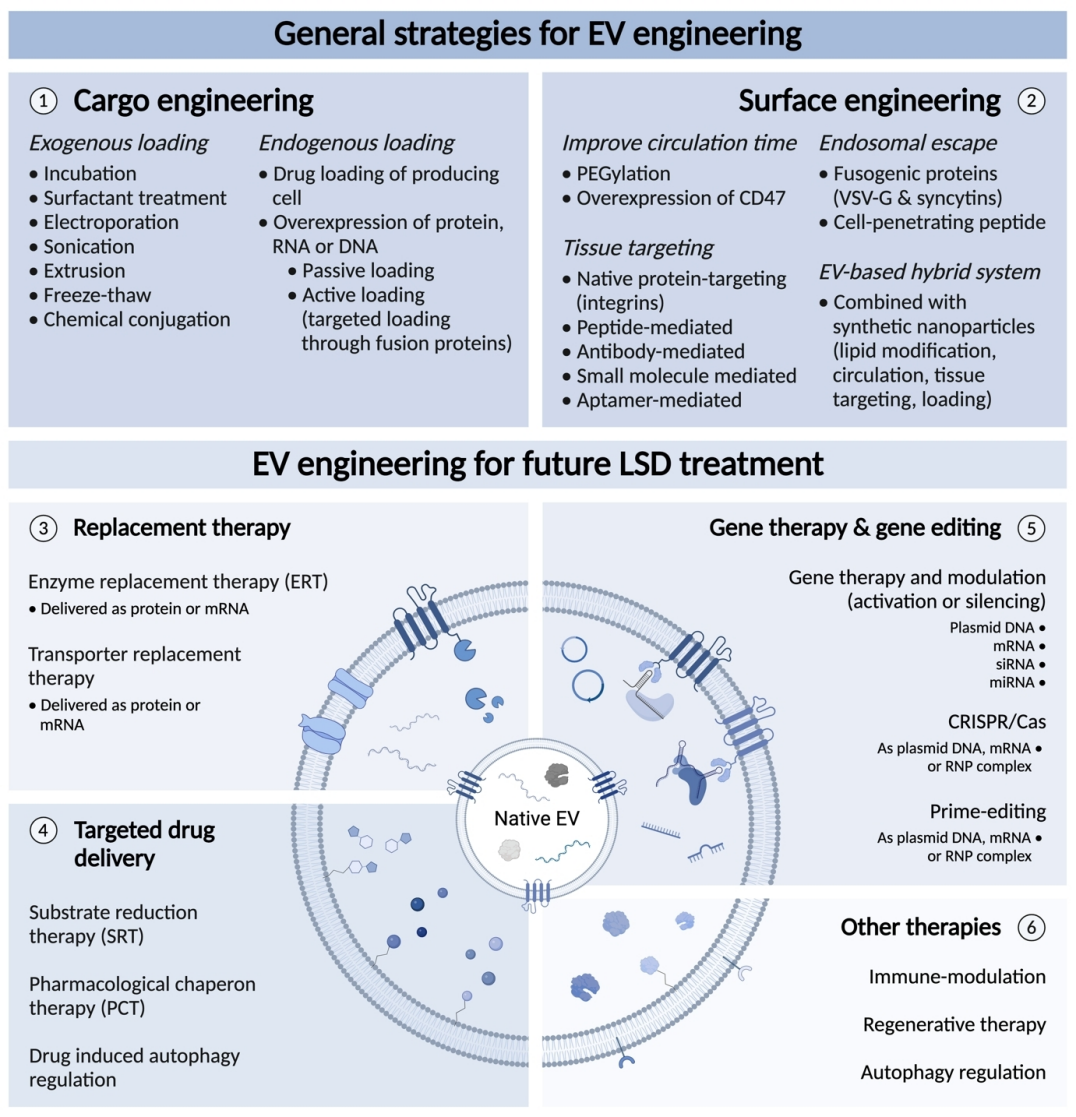
Future EV-based therapeutic options for the treatment of LSDs. Various EV engineering approaches and techniques can be used to optimize the therapeutic potential of EVs, including: (1) Cargo engineering, which can either be accomplished through exogenous loading techniques applied to the EVs post-isolation or endogenous loading techniques. In endogenous loading techniques, loading is accomplished through adaptation of the parental cell. Cargo can either be passively or actively loaded with the use of fusion proteins binding the cargo to EV-enriched proteins or transmembrane domains; (2) Surface engineering, which can improve circulation time of EVs, their tissue targeting potential and endosomal escape, which is needed for efficient intraluminal cargo delivery. Furthermore, EVs can be combined with synthetic nanoparticles. These hybrid systems can modulate the contents and functionality of both the particle surface and cargo. Future EV therapeutic modalities for LSDs potentially include; (3) Replacement therapies, which include enzyme-, and transporter-replacement therapy. These therapies can be loaded into EVs and delivered to target cells as mRNA or directly as protein; (4) Targeted drug delivery, for substrate reduction therapy (SRT), pharmacological chaperon therapy (PCT) and potentially drug induced autophagy regulation; (5) Gene therapy & gene editing, which includes loading of constructs such as plasmid DNA, mRNA, miRNA and siRNA for gene addition or modulation. Moreover, EVs could be engineered to contain CRISPR/Cas or Prime editing constructs in the form of plasmid DNA, mRNA or RNP complexes; and (6) Other therapies, which include unmodified or engineered EVs for adjuvant therapy, including immune-modulatory, regenerative therapy and autophagy regulation.

Efficient gene editing after EV mediated delivery of RNP complexes has already been demonstrated. Active loading can be obtained through direct or indirect fusion of the Cas9 protein to known EV markers. This was shown by the indirect loading approach of Ye *et al.*, who used CD63-GFP and Cas9-GFP nanobody fusion proteins that sufficiently loaded and delivered the complex^[184]^. Furthermore, Wang *et al.* proposed the use of ARRDC1, a protein required in plasma membrane budding together with known EV marker TSG101 to form ectosomes^[152]^. ARRDC1 interacts with proteins containing WW domains and recruits them into vesicles. Wang *et al. *fused either two or four WW domains to a Cas9 protein and showed that co-expression with ARRDC1 leads to sufficient loading of the RNP complex into EVs^[152]^. They went on to show functional gene editing upon delivery by knocking down GFP expression in their recipient cells using an anti-GFP sgRNA^[152]^. Recently, light‐induced dimerization loading was shown to be effective. In this approach, Osteikoetxea *et al.* used Cryptochrome 2 combined with either CD9 or a Myristoylation‐Palmitoylation‐Palmitoylation lipid modification^[185]^. This method provides controllable loading and release of the complex upon delivery^[185]^. In addition to loading of the complex through the Cas9 protein, RNA binding proteins such as HuR can increase complex loading by the addition of AU rich elements to the sgRNA^[159]^. At the same time, this strategy shows the potential of EV engineering in the delivery of other RNAs, including miRNA^[159]^ and mRNA^[152]^. Furthermore, the delivery of Cas9-sgRNA or prime editing complexes with EVs could be a major step towards a permanent cure for LSDs. Figure 2 gives an overview of the general EV engineering strategies and the potential of engineered EVs for future LSD treatment.

## CONCLUSION

As with any new class of therapeutic agents, EVs face several hurdles in their advancement into the clinic^[186]^. Optimization of loading efficiency, EV production, isolation, functional properties, purity, storage for potential off-the-shelf availability, as well as selection of the optimal cell source, is needed^[47]^. In addition, standardization of these processes’ clinical and industry-accepted validation must be developed for the regulatory approval of EV- diagnostics and therapeutics. A better understanding of EV biogenesis, uptake, cargo delivery and the normal *in vivo* journey will not only accelerate their therapeutic translation, but also improve their function in understanding LSD pathology and use as biomarkers. The EV field holds great potential and may one day contribute to diagnostics and the design of a new generation of smart vehicles for targeted delivery of drugs, ERT, RNA therapeutics and gene editing to manage or treat multisystemic LSDs.

## DECLARATIONS

### Copyright

© The Authors 2022.
